# Supplementary material for: A Systematic Review of Short-Term Outcomes of Minimally Invasive Thoracoscopic Surgery for Lung Cancer after Neoadjuvant Systemic Therapy
Source: Cancers (Basel). 2023 Aug 1;15(15):3908. doi: 10.3390/cancers15153908 (PMC10417737; doi:10.3390/cancers15153908)
Supplement: Supplementary file 1 [file cancers-15-03908-s001.zip › cancers-2512283-supplementary.pdf]

Supplemental Table S1: Complications per Study

Author	Year	Prolonged Air Leak N(%)		Arrythmia N(%)		Pneumonia N(%)		Wound Infection N(%)		Cardiac Complication N(%)		Atelectasis N(%)		ARDS N(%)		Pneumothorax or effusion N(%		Fistula N(%)		Empyema N(%)		Pulmonary Embollism N(%)		Respiratory Failure N(%)		Chylothorax N(%)	
		Open	VATS	Open	VATS	Open	VATS	Open	VATS	Open	VATS	Open	VATS	Open	VATS	Open	VATS	Open	VATS	Open	VATS	Open	VATS	Open	VATS	Open	VATS
**Huang [12]**	2013	NR	1 (2.4)	NR	NR	NR	NR	NR	1 (2.4)	NR	NR	NR	NR	NR	NR	NR	NR	NR	NR	NR	NR	NR	NR	NR	1 (2.4)	NR	1 (2.4)
**Yang, C [16]**	2016	6 (2.9)	3 (4.3)	7 (3.4)	7 (10.1)	4 (2.0)	2 (2.9)	NR	NR	NR	NR	NR	NR	NR	NR	NR	NR	NR	NR	NR	NR	NR	NR	1 (0.5)	0 (0)	NR	NR
**Kamel [13]**	2017	NR	NR	NR	NR	NR	NR	NR	NR	10 (13.5)	1 (2.5)	NR	NR	NR	NR	NR	NR	NR	NR	NR	NR	NR	NR	NR	NR	NR	NR
**Jeon [19]**	2018	4 (22.2)	2 (11.7)	4 (22.2)	3 (17.6)	1 (5.5)	1 (5.9)	2 (11.1)	0 (0)	NR	NR	NR	NR	1 (5.5)	1 (5.9)	NR	NR	NR	NR	NR	NR	0 (0)	2 (11.8)	NR	NR	NR	NR
**Yang, Z [15]**	2018	NR	1 (3.4)	NR	NR	NR	3 (10.3)	NR	1 (3.4)	NR	NR	NR	NR	NR	NR	NR	NR	NR	NR	NR	NR	NR	NR	NR	NR	NR	NR
**Cabanero sanchez [25]**	2022	18 (13.3)	7 (9.5)	13 (9.6)	2 (2.7)	9 (6.6)	1 (1.3)	5 (3.7)	0 (0)	NR	NR	8 (5.9)	2 (2.7)	1 (0.7)	0 (0)	2 (1.5)	0 (0)	1 (0.7)	0 (0)	2 (1.5)	0 (0)	1 (0.7)	0 (0)	NR	NR	NR	NR
**Dell'Amore[26]**	2022	19 (20.4)	11 (17.7)	NR	NR	NR	NR	NR	NR	NR	NR	NR	NR	NR	NR	NR	NR	NR	NR	NR	NR	NR	NR	NR	NR	NR	NR
**Jeon [28]**	2022	3 (0.8)	0 (0)	12 (3.4)	8 (22.8)	4 (1.1)	1 (2.8)	1 (0.3)	0 (0)	NR	NR	2 (0.6)	2 (5.7)	8 (2.3)	1 (2.8)	NR	NR	NR	NR	1 (0.3)	1 (2.8)	NR	NR	NR	NR	3 (0.8)	0 (0)
**Tian [30]**	2022	4 (5.6)	3 (5.4)	0 (0)	1 (1.7)	4 (5.6)	0 (0)	NR	NR	1 (1.4)	0 (0)	0 (0)	1 (1.8)	NR	NR	NR	NR	NR	NR	NR	NR	0 (0)	1 (1.8(	NR	NR	NR	NR
**Tong [31]**	2022	NR	4 (22.2)	NR	6 (33.3)	NR	1 (5.5)	NR	1 (5.5)	NR	NR	NR	3 (16.7)	NR	NR	NR	NR	NR	NR	NR	1 (5.5)	NR	1 (5.5)	NR	NR	NR	NR
**Yao [32]**	2022	NR	2 (18.2)	NR	1 (9.0)	NR	1 (9.0)	NR	NR	NR	NR	NR	NR	NR	NR	NR	NR	NR	NR	NR	NR	NR	NR	NR	NR	NR	NR
**Zhang [33]**	2022	1 (1.3)	5 (9.4)	3 (3.8)	1 (1.9)	8 (10.2)	2 (3.8)	NR	NR	NR	NR	NR	NR	NR	NR	NR	NR	NR	NR	NR	NR	NR	NR	2 (2.6)	0 (0)	1 (1.3)	0 (0)

VATS = video assisted thoracoscopic surgery, ARDS= acute respiratory distress syndrome, NR= not reported.
